# Effects of Single and Combined Ciprofloxacin and Lead Treatments on Zebrafish Behavior, Oxidative Stress, and Elements Content

**DOI:** 10.3390/ijms24054952

**Published:** 2023-03-03

**Authors:** Roxana Jijie, Emanuela Paduraru, Ira-Adeline Simionov, Caterina Faggio, Alin Ciobica, Mircea Nicoara

**Affiliations:** 1 Research Center on Advanced Materials and Technologies, Department of Exact and Natural Sciences, Institute of Interdisciplinary Research, Alexandru Ioan Cuza University of Iasi, Bd. Carol I, 700506 Iasi, Romania; 2 Doctoral School of Geosciences, Faculty of Geography and Geology, Alexandru Ioan Cuza University of Iasi, Bd. Carol I, 700505 Iasi, Romania; 3 Rexdan Research Infrastructure, “Dunarea de Jos” University Galati, 800008 Galati, Romania; 4 Department of Food Science, Food Engineering, Biotechnology and Aquaculture, “Dunarea de Jos” University Galati, 800008 Galati, Romania; 5 Department of Chemical, Biological, Pharmaceutical and Environmental Sciences, University of Messina, 98168 Messina, Italy; 6 Department of Biology, Faculty of Biology, Alexandru Ioan Cuza University of Iasi, Bd. Carol I, 700505 Iasi, Romania

**Keywords:** ciprofloxacin, lead, combined effects, behavior, biomarkers

## Abstract

Even though the toxic effects of antibiotics and heavy metals have been extensively studied in the last decades, their combined adverse impact on aquatic organisms is poorly understood. Therefore, the objective of this study was to assess the acute effects of a ciprofloxacin (Cipro) and lead (Pb) mixture on the 3D swimming behavior, acetylcholinesterase (AChE) activity, lipid peroxidation level (MDA—malondialdehyde), activity of some oxidative stress markers (SOD—superoxide dismutase and GPx—glutathione peroxidase), and the essential elements content (Cu—copper, Zn—zinc, Fe—iron, Ca—calcium, Mg—magnesium, Na—sodium and K—potassium) in the body of zebrafish (*Danio rerio*). For this purpose, zebrafish were exposed to environmentally relevant concentrations of Cipro, Pb, and a mixture for 96 h. The results revealed that acute exposure to Pb alone and in mixture with Cipro impaired zebrafish exploratory behavior by decreasing swimming activity and elevating freezing duration. Moreover, significant deficiencies of Ca, K, Mg, and Na contents, as well as an excess of Zn level, were observed in fish tissues after exposure to the binary mixture. Likewise, the combined treatment with Pb and Cipro inhibited the activity of AChE and increased the GPx activity and MDA level. The mixture produced more damage in all studied endpoints, while Cipro had no significant effect. The findings highlight that the simultaneous presence of antibiotics and heavy metals in the environment can pose a threat to the health of living organisms.

## 1. Introduction

In recent years, a wide and increasing variety of hazardous chemical compounds has been identified in water bodies due to different anthropogenic activities, such as industrial, agricultural, domestic, and healthcare processes [1]. Both inorganic and organic contaminants, such as heavy metals [2,3], pharmaceutical substances [4,5,6,7,8], personal care products [5,8,9], agrochemicals [10], microplastics [11,12], and nanoparticles [13] are often present in surface, ground, and drinking water. The presence of these pollutants in water systems is recognized worldwide as a serious threat to aquatic ecosystems and human health, and reducing their release into the environment is a priority for international actions.

Although some metals are essential elements (e.g., Fe, Na, K, Cu, Zn, Ca, and Mg) necessary for the normal biological functioning of organisms, most of these elements may be toxic even at low concentrations [14]. Thus, because of their high degree of toxicity, bioaccumulation tendency, and chemical stability, Pb, arsenic (As), cadmium (Cd), chromium (Cr), and mercury (Hg) have been included in the priority list of hazardous substances. For instance, the World Health Organization included Pb, As, Cd, and Hg within its ten chemicals of major public health concern [15]. Various studies have shown that chronic or acute Pb exposure can elicit detrimental effects on neurological, gastrointestinal, cardiovascular, hematologic, immunologic, and renal systems [16]. Despite significant progress in reducing Pb levels around the world, significant sources still exist. For example, high Pb levels ranging from 17 to 400 μg/L have been reported in Guiyu (South China), near an electronic waste area [17,18]. Concentrations of Pb in the Guangzhou segment of the Pearl River were in the 5–15 μg/L range [19,20]. Lead in amounts ranging from 32 to 3340 ppm has been measured in the bottom sediments of water bodies in the Upper Silesia region in southern Poland [21]. Rajaratnam et al. [22] evaluated Pb concentrations in the tap water of 95 new houses in the Sydney metropolitan area and found that 60% of homes tested above the Australian Drinking Water Guideline (10 μg/L) at the first draw sampling. A survey of lead content of tap water at the National Taiwan University campus revealed that approximately 10% of the samples collected showed Pb levels greater than 10 μg/L, with the highest value at 62.6 μg/L [23]. Recently, DeForest et al. [24] proposed acute and chronic biotic ligand model- (BLM) based ambient water quality criteria (AWQC) for Pb in freshwater, taking into account the influence of three water quality parameters: hardness, dissolved organic carbon, and pH. The acute toxic range was from 20 to 1000 μg/L, whereas the concentration of Pb with chronic toxicity ranged from 0.3 to 40 μg/L. It is worth highlighting that the prediction of metal toxicity in aquatic systems is mainly based on basic indicators, such as growth, reproduction, and survival, while their neurotoxicity has been rarely considered in risk assessment. Thus, it is necessary to address the neurotoxic effects induced by Pb exposure at environmentally relevant concentrations. Chen et al. [25] showed that exposure of embryos to lead acetate from 6 to 96 h post-fertilization (hpf) induces behavioral changes in zebrafish larvae (hyperactivity) and learning/memory impairments in adult zebrafish. Similar results were reported by Xu et al. [26], who found that the learning deficits produced by embryonic exposure to Pb persisted for at least three generations. Developmental Pb exposure (30 mM) significantly reduced the response rate to stimuli, inducing startle and escape behavior deficits in adult zebrafish [27]. Chronic exposure of adult zebrafish to a low concentration of lead chloride (PbCl_2_) induced memory deficit and abnormal exploratory behavior, characterized by elevated freezing and reduced exploration [28]. Moreover, biochemical assays revealed a reduction in AChE activity, serotonin and melatonin levels, as well as an increase in SOD activity and cortisol level in the brain in response to Pb treatment when compared with the control group. In agreement with a previous study, Zhu et al. [29] showed that Pb exposure at a concentration of 20 μg/L may enhance anxiety levels characterized by reduced locomotor activity. Li et al. [30] demonstrated that chronic exposure of adult male zebrafish to Pb at environmental concentration levels (1 μg/L, 10 μg/L, and 100 μg/L) impaired exploratory behaviors, inhibited spatial working memory, and disturbed light/dark preference. In addition, the produced effects on zebrafish locomotion were concentration-dependent and were associated with disturbed expression patterns of mRNA levels of key genes involved in neurodevelopment (gap43, syn2a, th, dat, and drd1b), neurotoxic effects (c-fos and gfap), and stress responses (tap, mt1, hsp70, and hsp90).

In general, environmental contaminants do not occur alone; both inorganic and organic compounds often coexist in water bodies, affecting organisms. For example, recent studies have reported the occurrence of both heavy metals and antibiotics in the environment [31,32]. In spite of antibiotics’ toxicity to aquatic life and their possible contribution to the spread and persistence of antimicrobial resistance, there are no standards for the regulation of antibiotics discharge in water systems. For instance, the macrolide antibiotics (e.g., erythromycin, clarithromycin, and azythromycin), amoxicillin, ciprofloxacin, sulfamethoxazole, and trimethoprim have been recommended to be included in the Watch Lists from European Union monitoring (Decision 2015/495/EU of 20 March 2015, Decision 2018/840/EU of 5 June 2018, Decision 2020/1161/EU of 4 August 2020, and Decision 2022/1307/EU of 22 July 2022). Ciprofloxacin (Cipro), an antibiotic of the fluoroquinolone class, is one of the most widely prescribed antibiotics in the USA, with an annual prescription rate of 173 per 1000 beneficiaries [33]. In Europe, consumption of ciprofloxacin, expressed as DDD per 1000 inhabitants per day, accounted for 48.6% in 2017 and 50.8% in 2009 of the total quinolone amount [34]. As a result of its widespread use, it is not surprising that Cipro reached the aquatic environment. The concentration of Cipro detected in surface waters varied between 7.7 and 5528 μg/L, while in wastewater treatment plant (WWTP) effluents the antibiotic was detected up to 341 μg/L [35]. According to published results, acute and chronic exposure of zebrafish at various stages of development (embryos, larvae, and adults) to antibiotics can induce behavioral impairments, oxidative stress, and histological alterations [36,37,38,39,40]. Tissue alterations in the liver of adult zebrafish were observed after 96 h exposure to low concentration of ciprofloxacin alone and in combination with paracetamol [38]. Shen et al. [39] revealed that high doses of Cipro (156–1949 mg/L) can induce cardiovascular toxicity. In addition, when zebrafish larvae were exposed to 2.34 and 9.38 mg/L of β-diketone antibiotics (DKAs), they had a higher basal swim rate than control groups at 120 hpf in both light and light-to-dark photoperiod experiments [40]. While the zebrafish exposed to 6.25 mg/L of β-diketone antibiotics (including ofloxacin, ciprofloxacin, enrofloxacin, doxycycline, chlortetracycline, and oxytetracycline) exhibited an anxiolytic behavior, characterized by increased travel distance, time spent in the upper portion of the test tank, and line crossings, the 25 mg/L DKAs treatment led to anxiety-like behavior [36]. A recent study has demonstrated that acute exposure to antibiotics by zebrafish may result in cognitive impairment and enhanced aggression behavior [41]. On the other hand, Plhalova et al. [42] reported no significant effects on growth and no histological changes in groups with Cipro concentrations up to 3 mg/L.

Results have demonstrated that metallothionein (MT) plays an important role in ameliorating the inter-cellular toxicity of metals, whereas the oxidative stress, apoptosis, tissue damage, and variations of trace element levels were identified to be involved in response to chemicals treatments [43,44,45,46].

The majority of toxicological studies have focused on effects induced by individual chemical exposure, rather than chemical mixtures. However, compared to single exposure, mixed contaminants may elicit combined toxic impacts on organisms, including additive, antagonistic, and synergistic effects. Therefore, further understanding of the joint toxic impacts of chemical mixtures is needed. For example, combined exposure to 10 mg/L Pb and paraquat triggered synergetic behaviour on ethoxyresorufin-O-deethylase (EROD), 7-benzyloxy-4-trifluoromethyl-coumarin-O-debenzyloxylase (BFCOD), glutathione-S-transferase (GST), and UDP-glucuronosyltransferase (UGT) activities in goldfish (*Carassius auratus*) livers [47]. Compared to the toxicities of individual metals, Cd and manganese (Mn) showed antagonistic effects, whereas the mixture of Cd + Pb, Mn + Pb, and Cd + Mn + Pb induced synergistic effects in *C. elegans* [48]. In contrast, an antagonistic effect on behavioral pattern was observed when co-exposing zebrafish larvae to lead and cadmium [49]. These results suggest that Pb treatment impaired locomotor activity, whereas Cd disrupted behavioral rhythms. Exposure to both Pb and decabromodiphenyl ether elicited synergistic effects on thyroid hormone levels in zebrafish [50] and impaired neuronal development in zebrafish larvae [29]. Combination treatment with Pb and repeated heat pulse has been proven to have synergistic effects on developmental neurotoxicity in zebrafish [51]. The concentration addition and independent action models are commonly used to evaluate and predict the effects of mixed contaminants [45,52]. The results have revealed that uptake of metals in a mixture with other chemicals may be suppressed or enhanced. For example, the ionic liquid M8OI suppressed Pb absorption in the fish brain [53], and Pb toxicity was ameliorated due to the competitive binding of Cd to active enzymes [43]. Meanwhile, the uptake of Cd was enhanced in the presence of Ni at concentrations above 0.1 μM [54]. A similar result was found by Miao et al. [20], who noted that titanium dioxide nanoparticles enhanced the bioconcentration of Pb, leading to the disruption of the endocrine and neuronal systems in zebrafish larvae. Moreover, the chemical structure of the organic pollutants may be altered in the presence of heavy metals, mediating their degradation or transformation. For example, by forming a Pb^2+^—cypermethrin complex through the CN group of insecticides [55] or an atrazine—Cd complex through five electron-donor atoms of herbicide [52], the bioavailability of chemicals may be reduced, acting together in an antagonistic manner.

Consequently, to determine the impairments induced by various chemical compounds against organisms, behavioral assays (e.g., light/dark preference test, novel tank test, T-maze test, 3D locomotion test, predator avoidance test, etc.) alongside biochemical, histological, and transcriptomic assays have been successfully applied [14,28,30]. According to the literature, the frequency and severity of alterations increased with enhancing exposure time and concentration [30,39]. In addition, interest in applying 3D tracking techniques to toxicology is growing, because it can identify subtle behavioral changes that may be neglected by 2D approaches. For example, Macrì et al. [56] showed that 2D results are flawed by under- and over-reporting of behavioral differences compared with 3D data. Several 3D tracking systems have been developed by using one camera and one mirror [57], or two mirrors [58], or two cameras positioned orthogonally [14] in order to create 3D swim paths and to analyze the behavioral endpoints (e.g., total distance traveled, average velocity, turn angle, angular velocity, meandering, freezing duration, time spent in the top/bottom, etc.). Among vertebrate models, over the past few years, zebrafish (*Danio rerio*) have gained increasing popularity for assessing the effects of environmental contaminants due to easy maintenance and manipulation, small size, high fecundity, rapid development, embryo transparency, and high sensitivity to chemical stressors. The strength of zebrafish is their complex behavior, including learning and memory, decision making, social interaction, and aggressive responses [59,60,61].

In this study, we aimed to evaluate the combined adverse impact Cipro and Pb may induce on zebrafish behavior, oxidative stress, and body elements content. For this purpose, adult wild-type, mix-gendered zebrafish were exposed to environmentally appropriate doses of Cipro and Pb as single chemicals and as a mixture for 96 h. To the best of our knowledge, the combined toxicity induced by Pb and Cipro exposure in adult zebrafish was not previously reported. In addition, although the toxicity of these contaminants alone was previously studied in aquatic organisms, little information is available concerning their behavioral effects.

## 2. Results and Discussion

The individual and joint effects of lead and ciprofloxacin on zebrafish behavior have been assessed by three-dimensional (3D) locomotion tracking. Because animal behavior is an integrated response to various biochemical and physiological processes, behavioral changes may be observed early, a short time after exposure, and at low concentrations; this can also provide a better understanding of joint toxicity between multiple contaminants.

**Figure 1:**
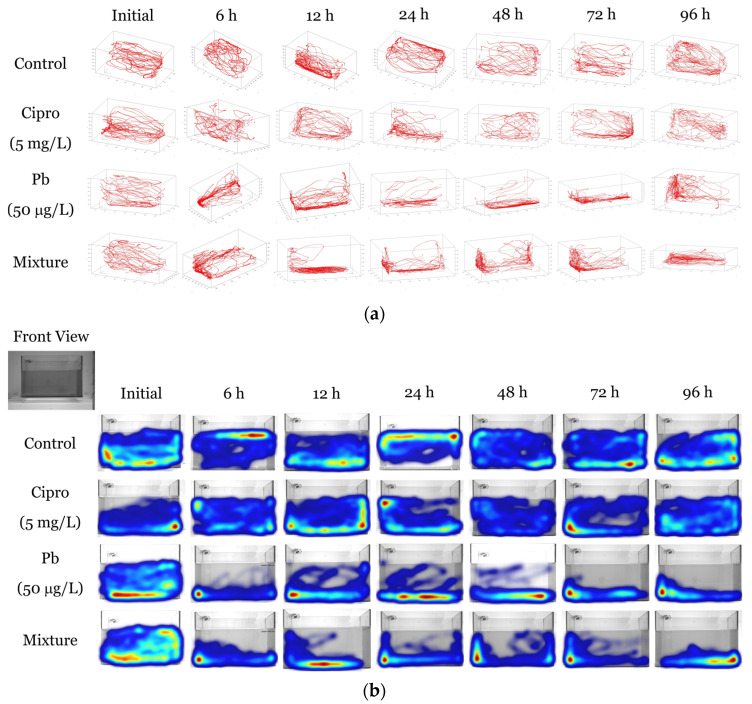

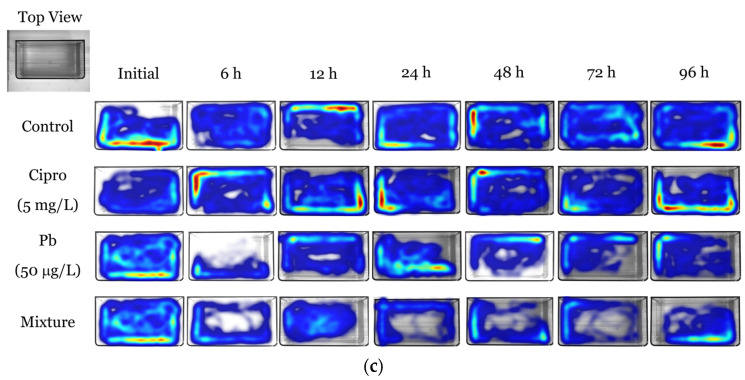
Behavioral patterns, including the representative 3D reconstructed swimming paths (**a**) and the 2D heat maps for *YZ* axis, front view (**b**) and *XY* axis, top view (**c**) of adult zebrafish from the control group and groups treated with 5 mg/L ciprofloxacin, 50 μg/L lead, and a mixture during 96 h of exposure (*n* = 10).

As shown in Figure 1a, exposure to Pb alone and in combination with ciprofloxacin significantly impaired zebrafish exploratory behavior by diminishing their swimming activity in the top part of the tank as compared with the control and Cipro groups. The administration of both nano-silica and reserpine [62], and of caffeine and fluoxetine alone [63], induced similar effects, decreasing zebrafish swimming activity in the upper part. In addition, a preference for staying near tank walls has been observed for zebrafish exposed to the binary mixture, known as thigmotaxis behavior. Similarly, treatment of zebrafish larvae with low concentrations of deltamethrin increased the time spent in close proximity to test tank walls [64]. In line with our results, Thi et al. [28] found that zebrafish locomotor activity and entries into the upper zone are reduced after the administration of 50 μg/L Pb, followed by a higher freezing time movement ratio. According to the literature, a longer time spent at the bottom of the tank and less time in the upper portion of the aquarium, preference for staying close to walls, and increased freezing behavior and counter-clockwise rotation illustrate a high anxiety level in zebrafish [37,65].

**Figure 2:**
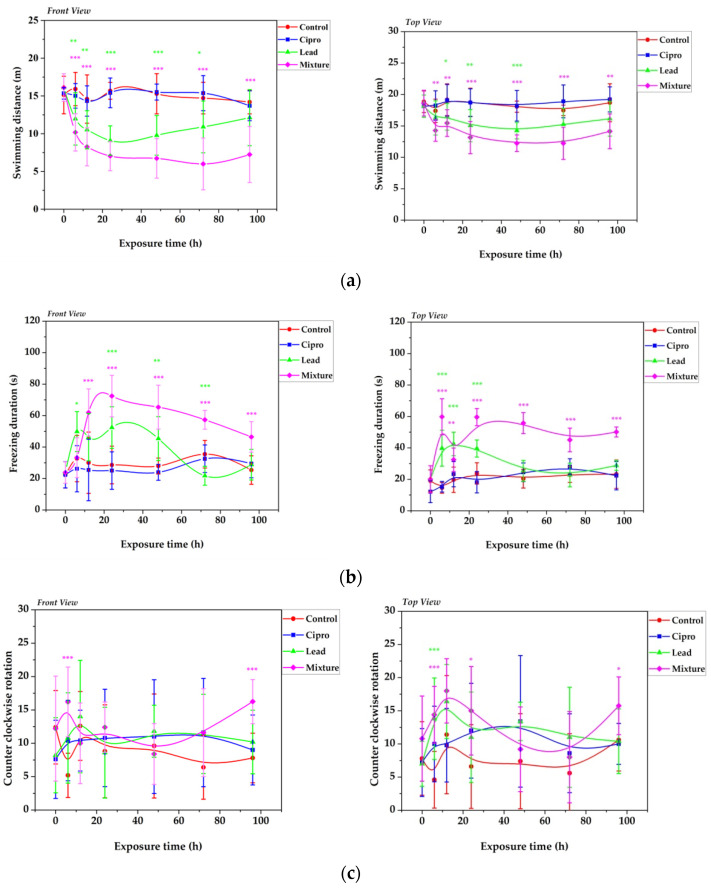
The locomotor behavior of adult zebrafish from control and treated groups with 5 mg/L ciprofloxacin, 50 μg/L lead, and a mixture for 96 h, which was measured by (**a**) the total distance traveled (m), (**b**) freezing duration, and (**c**) counter-clockwise rotation. Data are expressed as mean ± SD (*n* = 10) and were analyzed by one-way ANOVA followed by Dunnett’s post-hoc test. Statistically significant differences are denoted by * *p* < 0.05, ** *p* < 0.01, and *** *p* < 0.001.

There are no significant differences between the control and Cipro groups during the 96 h exposure period (Figure 2a–c), which is consistent with Petersen et al.’s results [41]. Changes in the swimming activity of zebrafish have been reported after 10 days of exposure to 10 mg/L ciprofloxacin [66]. In contrast, significant differences in all investigated behavioral endpoints have been observed for treated groups with Pb alone and in combination with an antibiotic, with more pronounced effects associated with the mixture. Exposure for 72 h to Pb alone and in combination with Cipro decreased the total distance moved in the side (YZ) plane by 26% and 59%, respectively, in contrast with the control, and by 12.8% and 30%, respectively, in the top (XY) plane. In addition, the behavioral impairments induced by lead seem to ameliorate from day 3 of the treatment. Similarly, Sehonova et al. [67] reported the ability of zebrafish juveniles to adapt to enrofloxacin in a short time period. On the other hand, both acute treatments increased the immobile mean time from an average of 10% of the time for the control to an average of 12% for the Pb-exposed group and 20% of the time for the mixture-treated group, respectively. These results are in agreement with previous findings related to triclosan [68] and alarm pheromone [63] treatments, which elevated freezing behavior and reduced exploration of zebrafish. Moreover, after 6 days of exposure to Pb (5–30 μg/L) with titanium dioxide nanoparticles (0.1 mg/L), the zebrafish showed slower locomotor behavior than those from control and single-exposure groups [20]. Similarly, common carp movement has been altered by chronic exposure of Pb at lower concentrations [53]. The authors linked neurobehavioral changes with increases in neurotransmitter dopamine levels and fish brain injuries. As illustrated in Figure 2c, combined administration of Pb and Cipro during the test significantly enhanced counter-clockwise rotation.

**Figure 3:**
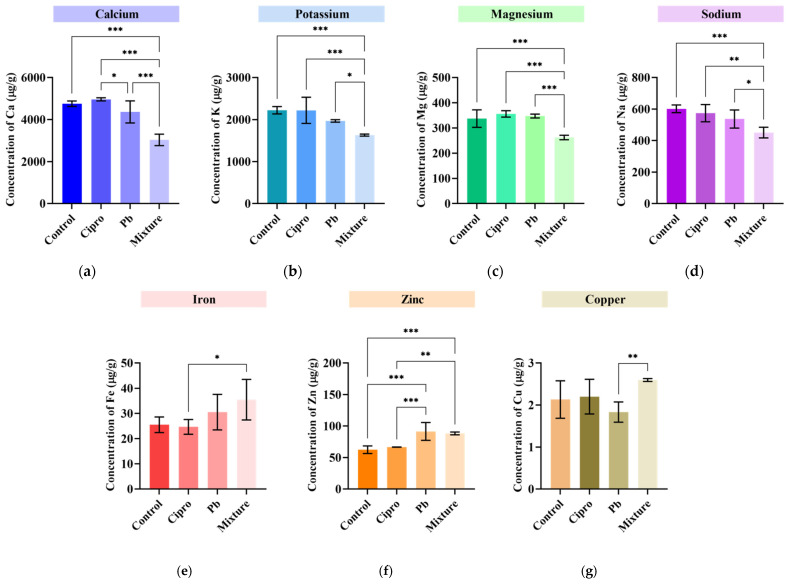
Concentrations of Ca (**a**), K (**b**), Mg (**c**), Na (**d**), Fe (**e**), Zn (**f**), and Cu (**g**) in the body of zebrafish of the control group and groups treated with 5 mg/L ciprofloxacin, 50 μg/L lead, and a mixture for 96 h. Data are expressed as mean ± SD (*n* = 5) and were analyzed by one-way ANOVA followed by Tukey’s post-hoc test. Statistically significant differences are denoted by * *p* < 0.05, ** *p* < 0.01, and *** *p* < 0.001.

Elements, such as Ca, K, Mg, Na, Cu, Fe, and Zn, are essential for the functioning of cellular enzymes and proteins involved in many physiological and metabolic processes [46]. For example, cations, such as Ca, K, Mg and Na, are essential elements for ensuring the homeostasis of intracellular and extracellular balance in the fish organism [69,70,71,72]. As can be observed in Figure 3a–g, the mixture of Cipro and Pb interfered with the fish’s ability to efficiently uptake the essential macro-elements Ca, K, Mg, and Na. The co-administration of contaminants decreased the concentration of Ca by 36%, of K by 26.8%, of Mg by 22%, and of Na by 25%, respectively, in the zebrafish body, a fact that clearly highlights the enhanced toxicity of the binary mixture. In line with our results, You et al. [73] reported an additive toxicity between florfenicol and Cu(II)/Cd (II) on *Synechocystis* sp. owing to a lack of interactions between them. In the case of the group exposed to antibiotics, it was found that Ca (4963.5 ± 71.3 μg/g) and Mg (355.8 ± 12.7 μg/g) had the highest concentration in the zebrafish body, but no significant difference was registered when compared with the control group. In cells, mitochondria are responsible for Ca and Mg regulation [74,75], and in some studies, it has been reported that antibiotic substances have the capacity to improve mitochondrial function [75]. Nevertheless, Kozieł et al. demonstrated in their study that exposure to 25 µg/L of ciprofloxacin limits the cells’ capacity to uptake Ca^2+^ [74]. However, the effect of antibiotics is dependent not only on their dosage but also on their type [76].

Cu and Fe are essential microelements involved in hemoglobin formation [77]. At the same time, it is well known that SOD activity relies on a specific catalytic metal ion, which could be copper (Cu/ZnSOD) [78,79]. As can be seen in Figure 3e–g, concomitant exposure to Pb and Cipro alleviated the concentration of Fe by 39%, of Zn by 41.3%, and of Cu by 21.8%, but the only significant difference when compared with the control group was found for Zn. This phenomenon can be associated with an intense SOD activity, which also registered the highest value in the binary mixture-exposed group, in order to cope with the generated oxidative stress. In addition, Fe overload in fish organisms has been previously reported as a response to oxidative stress [59]. Recently, Shaw et al. [80] showed a significant increase in the hepatic content of chromium, selenium, iron, manganese, calcium, sulfur, and magnesium but depletion of zinc, copper, and cobalt in a group treated with hexavalent chromium. After exposure to Zn under different pH values for 30 days, a decrease in Fe content and increase in Cu level were recorded in fish livers [81].

In summary, significant deficiencies of Ca, K, Mg, and Na contents, as well as an excess of Zn level, were observed in fish tissues after exposure to the Pb and Cipro binary mixture. Furthermore, these changes can potentially interfere with cellular antioxidant balance and trigger oxidative damage, which can be linked to the observed zebrafish exploratory behavior impairments.

**Figure 4:**
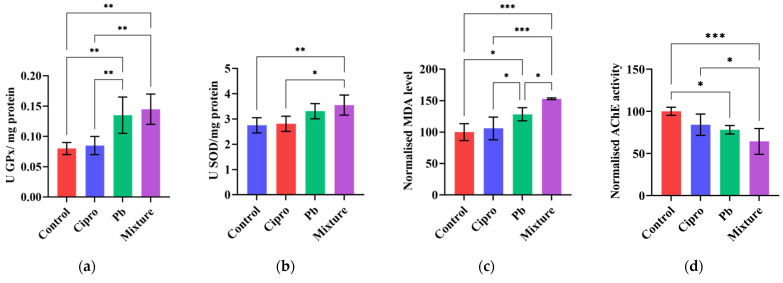
The responses of biomarkers, such as (**a**) GPx, (**b**) SOD, (**c**) MDA, and (**d**) AchE, following treatment with 50 μg/L Pb, 5 mg/L Cipro, and their mixture. Data are expressed as mean ± SD (*n* = 5) and were analyzed by one-way ANOVA followed by Tukey’s post-hoc test. Statistically significant differences are denoted by * *p* < 0.05, ** *p* < 0.01, and *** *p* < 0.001.

Oxidative stress occurs when the sensitive balance between the generation of reactive oxygen species (ROS) and ROS scavenging is disturbed by various stressors [12,82,83,84]. It has been reported that exposure to environmental contaminants may increase the production of ROS, leading to oxidative damage [82,85]. A relationship between the imbalance in the activities of enzymatic antioxidants (e.g., GPx, SOD, CAT, etc.) and histological, behavioral, and morphological alterations upon exposure to contaminants has been reported in the literature [14,35,86]. Likewise, the alteration of acetylcholinesterase (AChE) activity has been related to behavioral impairments [87]. In our study, exposure to lead alone and in combination with ciprofloxacin induced an oxidative stress response in zebrafish and a decrease in AchE activity in the brain over the 4-day exposure period, with more pronounced alterations with mixture treatment. As shown in Figure 4, the GPx activity and MDA level increased in both Pb alone and Pb + Cipro-treated groups, whereas a significant increase in SOD activity was obtained only for zebrafish exposed to the Pb—Cipro mixture. Ciprofloxacin treatment had no effect on biomarkers studied, which is consistent with behavioral results. In agreement with our results, Ramirez et al. [35] found an augmentation of SOD, CAT, and GPx activities, MDA level, protein carbonyl, and hydroperoxide contents in zebrafish embryos at 72 and 96 hpf for the binary mixture of ciprofloxacin and paracetamol. Similarly, treatment with Pb of the bivalve (*Chlamys farreri*) significantly reduced SOD, CAT, and GPx activities and induced high levels of MDA content [88]. Plhalova et al. [42] reported no significant differences between the control group and ciprofloxacin-treated groups (0.7–3000 μg/L) with respect to lipid peroxidation. Moreover, acute exposure to Pb alone and in mixture with Cipro reduced AChE activity in the zebrafish brain, also reported by other researchers [28,68,89,90]. Velázquez et al. [38] indicated that binary mixtures of ciprofloxacin and paracetamol caused more damage in all investigated endpoints in contrast with Cipro treatment.

Overall, our results contribute to a better understanding of the toxicity of heavy metals and antibiotics, individually and in a mixture. It can be concluded that the presence of heavy metals alone or in combination with antibiotics in water bodies may be harmful to aquatic species and their simultaneous presence may produce more damage. Cheng et al., based on the self-adaptive ability of organisms to polluted environments, hypothesized that Cd may impair defense capacity, therefore allowing more erythromycin bioaccumulation in tissues in the presence of metals [44]. However, further investigations are necessary to explain the mechanism that underlies the behavioral and biochemical responses to coexisting heavy metals and antibiotics.

## 3. Materials and Methods

### 3.1. Chemical Reagents

Lead standard solution Certipur (Pb, 1000 mg/L, 119,776), nitric acid 65% Suprapur^®^ (HNO3, 100,441), hydrogen peroxide 30% Perhydro^®^ (H_2_O_2_, 107,210), multi-element standard solution Certipur (111,355), acetylhiocholine iodide (ATCh, A5751), 5,5-dithio-2,2-nitrobenzoic acid (DTNB, 322,123), Bradford Reagent (B6916), Bovine Serum Albumin (A8022), ethanol EMSURE^®^ (159,010), phosphate buffered saline (P4417), tris hydrochloride solution (T2819), Lipid Peroxidation Assay Kit (MDA, MAK085), Superoxide Dismutase Assay Kit (SOD, 19,160), and Glutathione Peroxidase Cellular Activity Assay Kit (GPx, CGP1) were bought from Merk, Darmstadt, Germany. The ciprofloxacin liquid form (Cipro, 10 mg/mL) was obtained from a local pharmacy. The product was selected to simulate a real-life exposure situation and to avoid any possible conflict of interest by ensuring the manufacturer brand remains anonymous.

### 3.2. Zebrafish Maintenance and Exposure

The wild-type adult zebrafish (*Danio rerio*, AB strain, 8–12 month old, mass = 0.45 ± 0.05 g, body length = 28 ± 1 mm) were obtained from a local supplier. They were maintained in 65 L glass tanks at 26 ± 1 °C with a 12 h light/12 h dark cycle (lights on at 8:00 a.m.). The fish were fed twice daily with TetraMin Tropical Flakes, receiving a daily ration of ~1% of body weight. The aquarium was equipped with trickling filters and constantly aerated. The water parameters were determined and kept constant in all aquaria during the fish housing and treatment: pH 7.28 ± 0.1, conductivity 540 ± 3 μS/cm, salinity 0.25 ± 0.1, TDS 260 ± 5 mg/L, nitrite 0.03 ± 0.005 mg/L, nitrate 2.5± 0.5 mg/L.

**Figure 5:**
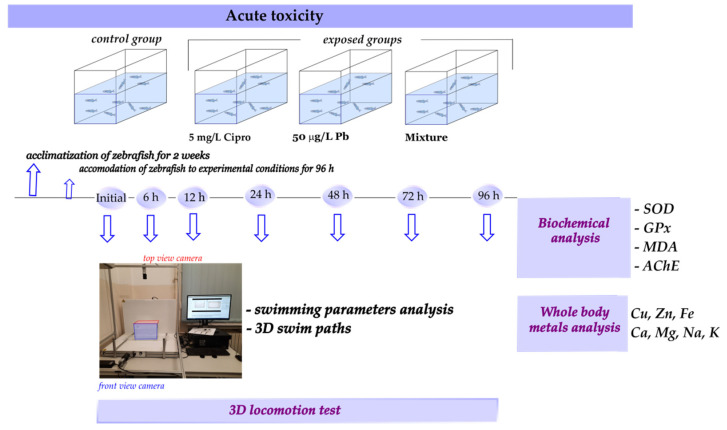
Experimental setup used to assess the potential effects of ciprofloxacin and lead treatments on zebrafish behavior, oxidative stress, and elements content.

Toxicity tests complied with OECD Guidelines for the Testing of Chemicals, Section 2: Effects on Biotic Systems, fish acute toxicity test No. 203. After acclimatization for 2 weeks, the zebrafish (*n* = 10 per group) were randomly separated into four groups, including a control group (fish were exposed to standard tank water), Cipro group (5 mg/L), Pb group (50 μg/L), and mixture group (5 mg/L of Cipro + 50 μg/L of Pb), as illustrated in Figure 5. The zebrafish were housed in trapezoid glass tanks (30 cm long × 16.5 cm wide × 20 high) with 5 L exposure solutions renewed daily and they were not fed 24 h prior to or during treatment. The concentration of lead and ciprofloxacin was selected based on previous studies [17,18,21,28,35,41]. In addition, the real concentration of Pb and Cipro following the single and combined treatments was determined with an atomic absorption spectrometer (HR-CS GF-AAS, ContrAA 700, Analytik Jena, Jena, Germany) following the method described by Jijie et al. [14] and with Specord 210 Plus spectrophotometer (Analytik Jena, Germany), as shown in Table 1. Briefly, the concentration of Pb and Cipro was evaluated from constructed calibration curve (y = 0.0085 + 0.0148 × for Pb and y = 0.0274 + 0.0247 × for Cipro), as shown in Figure S1 (Supplementary Materials). Three replications per sample were conducted for validating the nominal concentrations. The UV-Vis analysis revealed that there was no change in absorption bands intensities or positions from 0 to 24 h of treatments with 5 mg/L of Cipro alone or in mixture with 50 μg/L of Pb (Figure S2). Furthermore, for the mixture, within 24 h new peaks cannot be observed, indicating that the compounds did not interact with one another to form a new product. Similarly, Ding et al. [53] have shown that Pb and ionic liquid M8OI do not react within one day.

In order to acclimate them to handling stress, before starting the 3D locomotion test, during 96 h the animals were gently hand-netted from their experimental tank to a temporary tank. No mortality was recorded during the experimental period. Once the behavioral testing was completed, zebrafish from the control and the exposed groups were euthanized using hypothermic shock method (2–4 °C), according to animal welfare regulations [91].

### 3.3. Three-Dimensional (3D) Behavioral Testing

For the three-dimensional locomotion test, zebrafish (*n* = 10 per group) were placed individually in a temporary glass tank (30 cm long × 16.5 cm wide × 20 high) filled with 6 L of dechlorinated tap water. Then, following a 60 s habituation period, the locomotor activity was recorded with the Track3D module of EthoVisionXT 16 video tracking software (Noldus Information Technology, Wageningen, The Netherlands) for 4 min. The 3D tracking system has been described in our previous study [14]. The 3D locomotion tracking was recorded at *t* = 0 h (after the accommodation of animals to experimental conditions for 96 h) to set the baseline behavior of fish (presented as initial behavior), 6 h, 12 h, 24 h, 48 h, 72 h, and 96 h for each individual and each experimental condition (5 mg/L of Cipro, 50 μg/L of Pb and their mixture). In order to gain insight into the acute toxicity induced by Cipro and Pb alone and in combination, the following behavioral endpoints were analyzed: the total distance traveled (m), freezing duration (s), and counterclockwise rotation.

### 3.4. Biochemical Assays

Briefly, frozen tissues were placed into a KIMBLE Dounce tissue grinder set (Sigma) with 10 volumes of ice-cold phosphate buffered solution and then centrifuged at 5500 rpm for 15 min (4 °C). Then, the supernantants were used for evaluation of oxidative stress. On evaluation of oxidative stress, the following antioxidant enzymes were determined: SOD and GPx, as well as the lipid peroxidation (MDA) level. Activities of SOD and GPx and MDA levels were determined colorimetrically using kits (Merck, Darmstadt, Germany) following the manufacturer’s instructions. The results were normalized to the total protein content determined with the Bradford method using bovine serum albumin as standard, while MDA level was normalized as percentage of control values.

AChE activity was measured according to a previously reported protocol [87].

### 3.5. Determination of Whole Body Ions

Before analysis, all samples (*n* = 5 per group) were previously digested (~1 g) in a microwave-assisted pressure digestion system (TOPwave, Analytik Jena, Germany), by using a mixture of 5 mL nitric acid (HNO_3_ 65% Suprapur, Merck, Germany) and 2 mL hydrogen peroxide (H_2_O_2_ 30% Emsure, Merck, Germany). Further on, the mineralized samples were transferred in clean polyethylene flasks (50 mL volume) and diluted with ultrapure water. Calcium, potassium, magnesium, sodium, iron, and zinc were quantified by using the flame atomic absorption spectrometry (FAAS) technique. In case of copper determination, the graphite furnace atomic absorption spectrometry technique was used. All measurements were carried out by using ContrAA 700 by Analytik Jena Germany. For the calibration of the equipment, a multi-element standard solution (1000 mg L^−1^) was used. The methods’ accuracy was validated by using a reference material for fish muscle (ERM-BB422), certified by the EU Joint Research Center Institute for Reference Materials and Measurements. All concentrations were expressed as μg/g wet weight ± SD.

### 3.6. Statistical Analysis

Initially, the distribution of the data was evaluated for normality by the Shapiro–Wilk test. Afterward, one-way ANOVA followed by Tukey’s or Dunnett’s post-hoc tests were used, depending on the need to compare each exposure group to the control group or the mean of each column with the mean of every other column. All statistical analyses were conducted with Graph Pad Prism v.9.0 (GraphPad Software, San Diego, CA, USA). The results were presented as mean ± standard deviation (SD) and for all comparisons, *p* ≤ 0.05 was considered significant. Plots were generated in both Graph Pad Prism v.9.0 and OriginPro v.9.3 (2016, OriginLab Corporation, Northampton, MA, USA).

## 4. Conclusions

In summary, the short-term exposure of adult zebrafish to Pb alone and in combination with Cipro at environmentally appropriate doses induced abnormal behavior, characterized by elevated freezing duration and reduced swimming activity. Moreover, a significant depletion in the zebrafish body content of Ca, K, Mg, and Na, as well as an augmentation of Zn, have been determined in the treated group with Pb and Cipro mixture. Likewise, the biochemical assays revealed a reduction in AChE activity in the zebrafish brain and an increase of GPx activity and MDA level in the skeletal muscle, following treatment with Pb alone and in combination with Cipro. In all investigated endpoints, the mixture treatment produced more damage, while no significant differences were measured for the Cipro group. Therefore, the chronic or acute evaluation of a single pollutant does not give a realistic view, and the combined effects of various stressors against aquatic organisms should be assessed. Future in-depth investigations are required to illuminate the mechanisms underlying single and joint toxic effects.

## Figures and Tables

**Table 1:** Nominal and measured concentrations of Cipro and Pb in tank water following single and combined treatments. Data are shown as mean ± SD in three replicates.

Contaminants		Cipro	Pb	Mixture	
				Cipro	Pb
Nominal concentrations		5 mg/L	50 μg/L	5 mg/L	50 μg/L
Measured concentrations	t_0_ ^1^	4.71 + 0.41	49.1 + 2.5	4.83 + 0.31	49.8 + 1.9
	t_4_ ^2^	4.67 + 0.35	49.3 + 2.1	4.69 + 0.45	49.5 + 1.8
	t_24_ ^3^	4.63 + 0.45	48.9 + 2.6	4.65 + 0.35	49.1 + 2.5

^1^ Measured concentrations immediately after administration. ^2^ Measured concentrations after 4 h. ^3^ Measured concentrations after 24 h, before the renewal of exposure media.

## Data Availability

Not applicable.

## Supplementary Materials

The following supporting information can be downloaded at: https://www.mdpi.com/article/10.3390/ijms24054952/s1.
