# Supplementary material for: Effects of Single and Combined Ciprofloxacin and Lead Treatments on Zebrafish Behavior, Oxidative Stress, and Elements Content
Source: Int J Mol Sci. 2023 Mar 3;24(5):4952. doi: 10.3390/ijms24054952 (PMC10003324; doi:10.3390/ijms24054952)
Supplement: Supplementary file 1 [file ijms-24-04952-s001.zip › ijms-2195593-supplementary.pdf]

International Journal of
Molecular Sciences

MDPI

Supplementary Materials

Effects of Single and Combined Ciprofloxacin and Lead Treatments on Zebrafish Behavior, Oxidative Stress, and Elements Content

Roxana Jijie [1,*], Emanuela Paduraru [2], Ira-Adeline Simionov [3,4], Caterina Faggio [5], Alin Ciobica [6] and Mircea Nicoara [2,6]

[1] Research Center on Advanced Materials and Technologies, Department of Exact and Natural Sciences, Institute of Interdisciplinary Research, Alexandru Ioan Cuza University of Iasi, Bd. Carol I, 700506 Iasi, Romania
[2] Doctoral School of Geosciences, Faculty of Geography and Geology, Alexandru Ioan Cuza University of Iasi, Bd. Carol I, 700505 Iasi, Romania; emanuelapaduraru19@yahoo.com (E.P.); mirmag@uaic.ro (M.N.)
[3] Rexdan Research Infrastructure, "Dunarea de Jos" University Galati, 800008 Galati, Romania; ira.simionov@gmail.com
[4] Department of Food Science, Food Engineering, Biotechnology and Aquaculture, "Dunarea de Jos" University Galati, 800008 Galati, Romania
[5] Department of Chemical, Biological, Pharmaceutical and Environmental Sciences, University of Messina, 98168 Messina, Italy; caterina.faggio@unime.it
[6] Department of Biology, Faculty of Biology, Alexandru Ioan Cuza University of Iasi, Bd. Carol I, 700505 Iasi, Romania; alin.ciobica@uaic.ro
* Correspondence: roxana.jijie@uaic.ro

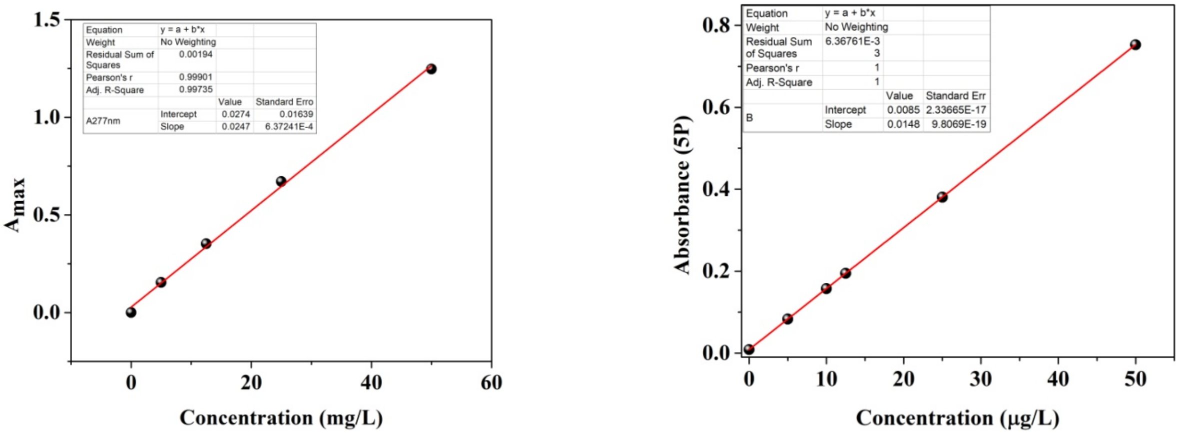

(a) (b)

Figure S1. Calibration curves for (a) ciprofloxacin and (b) lead.

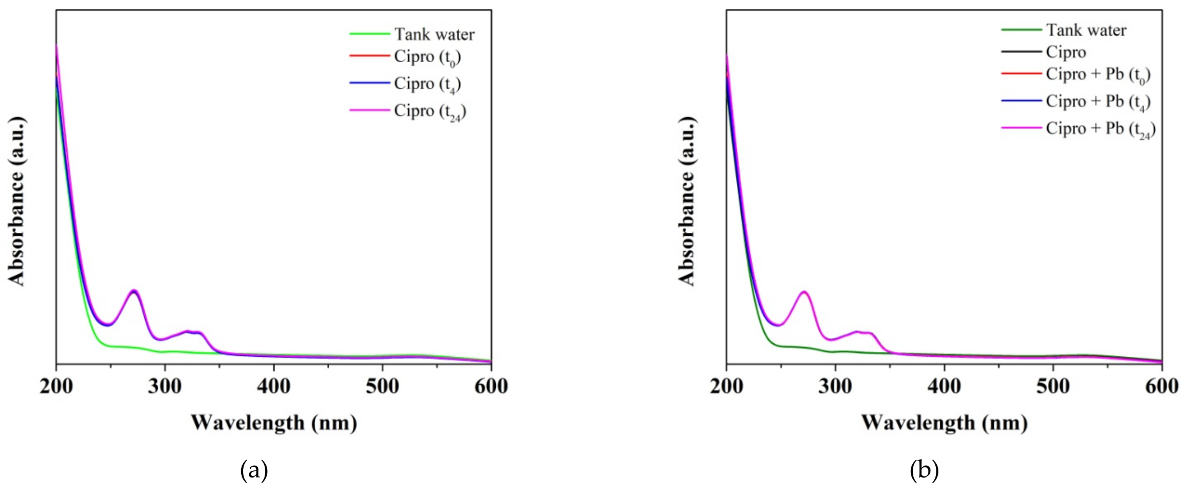

Figure S2. UV-Vis absorption spectra of Cipro alone (a) and in mixture with lead (b) for freshly prepared exposure solutions (black and red curves), after 4 h (blue curve) and 24 h (pink curve) before the renewal of exposure media.
